# Stroke-Like Presentation Following Febrile Seizure in a Patient with 1q43q44 Deletion Syndrome

**DOI:** 10.3389/fneur.2016.00067

**Published:** 2016-05-04

**Authors:** J. Elliott Robinson, Stephanie M. Wolfe, Kathleen Kaiser-Rogers, Robert S. Greenwood

**Affiliations:** ^1^School of Medicine, University of North Carolina School of Medicine, Chapel Hill, NC, USA; ^2^Division of Child Neurology, Department of Neurology, University of North Carolina School of Medicine, Chapel Hill, NC, USA; ^3^Department of Pathology and Laboratory Medicine, University of North Carolina School of Medicine, Chapel Hill, NC, USA

**Keywords:** hemiconvulsion–hemiparesis–epilepsy, 1q43q44 deletion, COX20, FAM36A, mitochondrial

## Abstract

Hemiconvulsion–hemiplegia–epilepsy syndrome (HHE) is a rare outcome of prolonged hemiconvulsion that is followed by diffuse unilateral hemispheric edema, hemiplegia, and ultimately hemiatrophy of the affected hemisphere and epilepsy. Here, we describe the case of a 3-year-old male with a 1;3 translocation leading to a terminal 1q43q44 deletion and a terminal 3p26.1p26.3 duplication that developed HHE after a prolonged febrile seizure and discuss the pathogenesis of HHE in the context of the patient’s complex genetic background.

## Case Report

The patient is a 3-year-old male who was found in bed experiencing a tonic–clonic seizure with left eye deviation. The duration of the seizure was unknown given that his mother had last seen him at least 3–4 h prior to the event. The patient was noted by his mother to be febrile to 102.5°F prior to witnessed seizure activity; upon arrival of EMS, his temperature was 100.5°F. He was transported to a local hospital, seizures were acutely controlled with lorazepam and levetiracetam, and he was transitioned to his home zonisamide regimen. Initially, the patient was assumed to have Todd’s paralysis, but when he failed to return to baseline, an MRI was performed on day 2 that showed thinned and truncated corpus collosum, diffusely increased T2/FLAIR signal intensity throughout the cortices of the left hemispheric gray matter with associated restriction diffusion, left hemispheric edema, 6.8 mm midline shift, effacement of the left ventricle, and dilation of the right ventricle. He was transferred to our tertiary hospital for neurosurgical management. At the time of admission, the patient was minimally responsive and displayed right-sided hemiparesis and gaze deviation, increased tone bilaterally, left papilledema, left orbital swelling, and asymmetric pupils who were reactive to light. vEEG was significant for diffuse slowing of the L hemisphere without epileptiform discharges.

**Figure 1:**
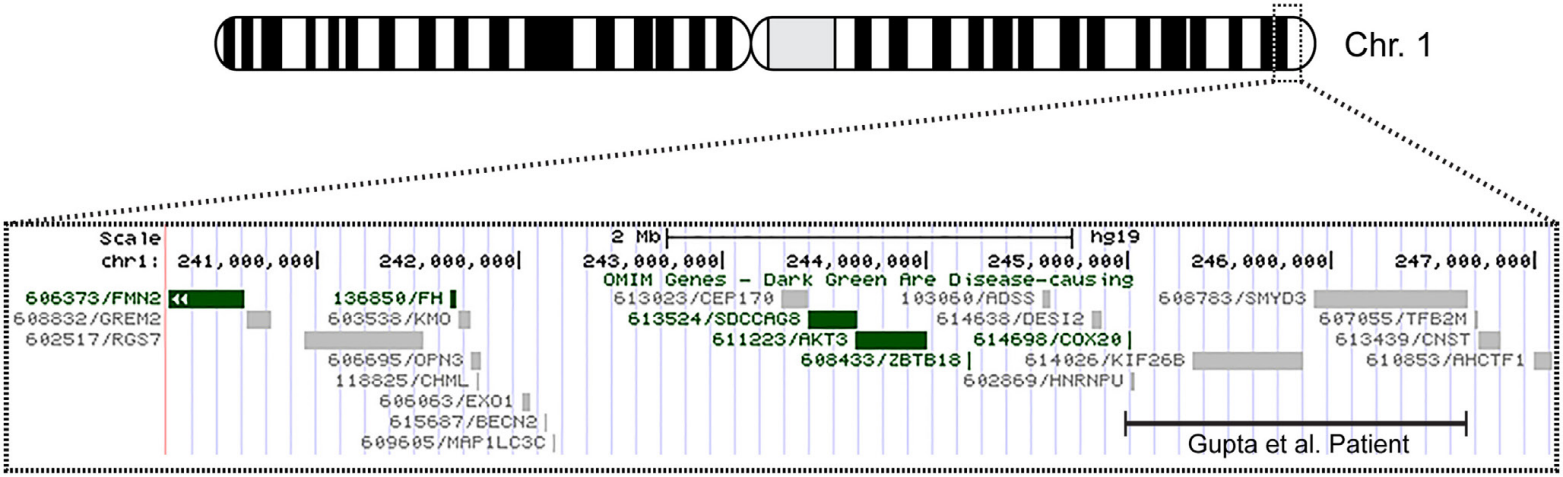
**Overview of patient’s chromosome 1 deletion**. Chromosome 1 ideogram displaying our patient’s 6.9 Mb deletion and the deletion observed in the patient described by Gupta et al. (1). The OMIM genes common to both deletions include *COX20 (FAM36A)*, *HNRNPU*, *HNRNPU-AS1*, *EFCAB2*, *KIP26B*, and *SMYD3*.

The patient’s past medical history was significant for hypotonia, global developmental delays, febrile seizures, breath holding spells, small stature, and patent foramen ovale. The child was born at 41 weeks to non-consanguinous parents following an uncomplicated delivery. He was found to have IUGR, a two vessel cord, ear indentations, branchial cleft cyst, small phallus, and thrombocytopenia. The patient developed apnea and staring spells during the first week of life, and MRI performed on postnatal day 8 did not reveal structural abnormalities or infarcts. Subsequent magnetic resonance angiography (MRA) demonstrated an asymmetrically small right transverse sinus that was patent. Karyotype and chromosome microarray analysis were performed, and the patient was found to carry an unbalanced 1;3 translocation that results in a 6.9-Mb deletion of the distal long arm of chromosome 1 containing 22 genes and an 8.6-Mb duplication of the distal short arm of chromosome 3 that contains 15 genes [46,XY,t(1;3)(q43;p26.1). arr[hg18] 1q43q44(240,287,612-247,190,999)x1,3p26.3p26.1(35,332-8,625,632)x3] (Table 1). Suspecting an association with the patient’s known chromosomal abnormality, the literature was reviewed, and another patient with similar symptoms and a smaller overlapping deletion involving 1q43q44 was identified (1) (Figure 1).

**Table 1:** **Deleted and duplicated OMIM genes resulting from an unbalanced t(1;3)(q43;p26.1)**.

Gene deletions			Gene duplications		
Gene	Locus	OMIM ID	Gene	Locus	OMIM ID
*FMN2*	1q43	606373	*CHL1*	3p26.1	607416
*GREM2*	1q43	608832	*CNTN6*	3p26-p25	607220
*RGS7*	1q43|1q23.1	602517	*CNTN4*	3p26.3	607280
*FH*	1q42.1	136850	*IL5RA*	3p26-p24	147851
*KMO*	1q42-q44	603538	*TRNT1*	3p25.1	612907
*OPN3*	1q43	606695	*CRBN*	3p26.2	607417
*CHML*	1q43	118825	*SETMAR*	3p26.1	609834
*EXO1*	1q42-q43	606063	*SUMF1*	3p26.1	272200
*MAP1LC3C*	1q43	609605	*ITPR1*	3p26.1	117360
*CEP170*	1q44	613023	*EGOT*	3p26.1	611662
*SDCCAG8*	1q43	613524	*BHLHE40*	3p26	604256
*AKT3*	1q44	603387	*EDEM1*	3p26.1	607673
*ZBTB18*	1q44	608433	*GRM7*	3p26.1-p25.1	604101
*ADSS*	1q44	103060	*LMCD1*	3p26-p24	604859
DESI2	1q44	614638	*LINC00312*	3p25.3	610485
COX20 (FAM36A)	1q44	614698			
HNRNPU	1q44	602869			
KIF26B	1q44	614026			
SMYD3	1q44	608783			
*TFB2M*	1q44	607055			
*CNST*	1q44	613439			
*AHCTF1*	1q44	610853			

*The genes common to the deletion in this patient and that reported in Gupta et al. include the OMIM genes highlighted above COX20 (FAM36A), HNRNPU, KIP26B, and SMYD3, as well as HNRNPU-AS1 and EFCAB2*.

**Figure 2:**
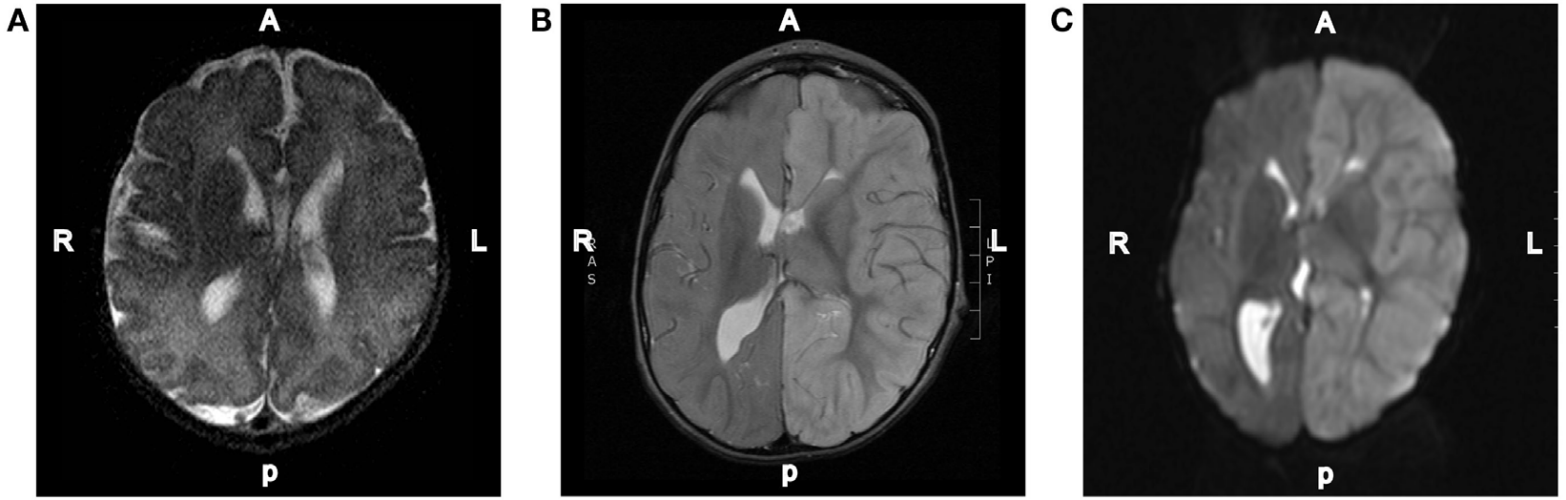
**Representative MRI images**. **(A)** T2-weighted axial brain MRI done of this patient at 3 days of age. **(B)** T2-weighted axial brain MRI done on day 2 of the hospital admission showing diffuse increased T2 signal intensity throughout the left hemispheric gray matter. **(C)** Diffusion-weighted axial brain MRI done on day 2 of the hospital admission showing diffuse left hemispheric cerebral edema.

Repeat MRI was performed on day 4 and showed worsening left-to-right subfalcine herniation anteriorly, patent arterial and venous supply, and left-sided diffusion restriction (Figure 2). Magnetic resonance spectroscopy (MRS) showed associated significant increase in lactate within the left cerebral hemisphere with decreased *N*-acetylaspartic acid, choline, and creatinine. A smaller but elevated lactate signal was observed in the right hemisphere. Due to worsening left hemispheric edema, the patient was started on intravenous mannitol on the third day of admission and transitioned to enteral sodium chloride 24 h later. Head CT on the sixth day of admission indicated that hemispheric swelling had stabilized, and repeat MRI/MRS on day 8 was essentially unchanged. Laboratory testing on this day revealed decreased total and free carnitine levels (14 and 16 nmol/mL, respectively); venous lactate was within normal limits (0.8 mmol/L). Given the patient’s history, clinical presentation, and MRI findings, he was diagnosed with hemiconvulsion–hemiplegia–epilepsy syndrome (HHE). The patient was seen in clinic approximately 2 months later after the completion of inpatient rehabilitation and displayed dense right hemiparesis with the ability to sit alone without support. His mother reported that his vocalizations and cognitive function had returned to baseline, and he was attempting to crawl again. At this time, the patient had not experienced a seizure since the inciting event.

## Discussion

Hemiconvulsion–hemiplegia–epilepsy syndrome is a rare outcome of prolonged focal status epilepticus that typically occurs in children under 4 years of age, in the context of febrile illness. While the incidence is unknown, it has been reported to have declined during the last several decades (2). A 2012 review of all published cases by Auvin and colleagues (3) reveal that approximately 30% of cases have precipitating factors (e.g., cortical dysplasia, viral infection, and known genetic polymorphisms), while the rest are idiopathic in nature. Clinically, HHE is characterized by prolonged unilateral clonic seizure followed by diffuse unilateral hemispheric edema and the development of hemiplegia (4). Cytotoxic edema in HHE may be radiographically similar to acute ischemic stroke, except that it is not limited to discrete vascular territories and is associated with normal MRA. The resolution of hemispheric edema is usually followed by global cerebral hemiatrophy, recurrent epilepsy, and persistent hemiplegia. Early neuroradiographic findings include increased ipsilateral T2- and diffusion-weighted signals, decreased apparent diffusion coefficient (ADC), and, in some cases, midline or temporal lobe herniation (3). These findings are associated with increased lactate and reduced *N*-acetyl acetate (NAA) signals on MRS (3, 5). While the acute phase of HHE is characterized by ictal discharges on EEG, slow waves and spikes followed by higher amplitude delta wave slowing may be seen in the affected hemisphere following the seizure (3). Several postmortem pathological examinations of brain parenchyma from affected individual have been mixed; findings include diffuse cortical scarring, edema and necrosis of cortical layers III and V, axonal damage, and spongiosis within the temporal lobe (6–8).

The pathophysiology of HEE has not been elucidated but may involve neuronal injury secondary to excitotoxicity and inflammatory changes triggered by prolonged hemiconvulsion (3). In this framework, neuronal energy metabolism is perturbed during the ictal event (9) and leads to excitoxic cell injury that is worsened by the cytokine-mediated inflammatory milieu caused by concurrent febrile illness (4). Genetic factors, such as channelopathies secondary to mutation within *SCN1A* (10) or *CACNA1A* (11) or pro-thrombotic states due to the presence of factor V Leiden (12), protein S deficiency (12, 13), or the *MTHFR* C677T polymorphism (12), may predispose the patient to HHE by increasing the duration of ictal activity, lowering the febrile seizure threshold, and/or exacerbating oxidative stress during seizure activity. To date, it unknown why seizures are restricted to a single hemisphere, although it has been hypothesized that the slow kinetics of corpus callosum development may limit seizure generalization (14). Another important unanswered question is whether if specific infectious agents preferentially predispose to HHE. For example, infection with human herpes virus-6 or -7 (HHV-6 and HHV-7, respectively) has been linked to febrile seizure in the general population (15, 16); yet, there has only been one published case of HHV-7 detected in the CSF of an HHE patient (17).

For this patient, cytogenetic analysis revealed a 1q43q44 deletion and a 3p26.1p26.3 duplication. Analysis of the deleted portion reveals 22 missing genes on chromosome 1 (Table 1), several of which appear to be medically relevant. *AKT3*, which codes for a serine–threonine kinase that is active during brain development, has been associated with microcephaly, while abnormalities of the corpus collosum may involve *ZBTB18* (*ZNF238)*, whose product is a C2H2-type zinc finger protein that is expressed during embryogenesis and functions as a transcriptional repressor (18). Analysis of the duplication reveals three doses of 15 genes (Table 1). 3p26 duplications have not been widely described in the literature and are generally not associated with seizure. However, based on a single patient with developmental disability and epilepsy, as well as a 3p26.3 duplication that includes the *CHL1* gene that codes for neural cell adhesion molecule L1-like protein, it has been proposed that this gene is a dosage sensitive one that may predispose to abnormal cognitive development (18).

Only five 1q44 deleted genes (Table 1) are the same in our patient and the patient with HHE reported by Gupta et al. (1), and only two genes, *COX20* (*FAM36A*) and *HNRNPU*, have been linked to intellectual disability and epilepsy (18, 19). *HNRNPU* codes for heterogeneous nuclear ribonucleoprotein U, which is a ribosomal protein involved in pre-mRNA processing during development (18). The FAM36A protein, a human ortholog of COX20, facilitates assembly of cytochrome *c* oxidase (COX; Complex IV of the electron transport chain), and mutant FAM36A can result in decreased COX expression in patient fibroblasts and may limit mitochondrial capacity for oxidative phosphorylation (20, 21). Given the role of *COX20* in proper cellular expression of COX and the observation that mitochondrial encephalopathy, lactic acidosis, and strokes (MELAS) is noted for producing rapid onset of MRI signal changes associated with cytotoxic edema (e.g., decreased ADC) without a vascular distribution like the changes seen in our patient, it is possible that mitochondrial dysfunction related to *COX20* deletion may have exacerbated the patient’s symptoms. Metabolic workup during admission, however, was equivocal, and the patient’s presentation likely had a multifactorial etiology.

Data from recent sequencing and copy number variation studies suggest that heterozygous loss of any one of the five OMIM genes deleted in both our patient and the patient described by Gupta et al. is not sufficient to cause HHE. In each case, multiple sequence variants likely to result in loss of function (i.e., nonsense, frameshift, or splice site changes) were identified in the Exome Aggregation Consortium (ExAC) database. With the exception of KIF26B and SMYD3, no exon containing deletions involving these genes individually or *en masse* were identified in either the Children’s Hospital of Philadelphia (CHOP) database or the Database of Genomic Variants (DGV) for benign copy number variation. While these data suggest that heterozygous loss of any one of these genes is compatible with normal development, it is conceivable that haploinsufficiency may contribute to the development of HHE, possibly in conjunction with sequence and/or copy number variants located within the complementary genes on the non-deleted homolog or elsewhere within the genome. Further studies, including obtaining a muscle biopsy to assay COX function and sequencing non-deleted genomic regions on the normal chromosome 1 homolog for deleterious mutations, will be necessary to elucidate the etiology of this patient’s presentation.

## Ethics Statement

The patient’s mother provided verbal and written consent for use of the patient’s personal health information in this case report in order to disseminate knowledge of the patient’s presentation to the broader medical community.

## Author Contributions

JR and SW wrote the manuscript, JR prepared the figures, KK-R completed genomic analysis, and RG and KK-R edited the final manuscript.

## Conflict of Interest Statement

The authors declare that the research was conducted in the absence of any commercial or financial relationships that could be construed as a potential conflict of interest.

## References

[1] Gupta R Agarwal M Boqqula VR Phadke RV Phadke SR. Hemiconvulsion-hemiplegia-epilepsy syndrome with 1q44 microdeletion: causal or chance association. Am J Med Genet A (2014) 164A(1):186–9. 10.1002/ajmg.a.36198 24214579

[2] Roger J Dravet C Bureau M Unilateral seizures: ­hemiconvulsions-hemiplegia syndrome (HH) and hemiconvulsions-hemiplegia-epilepsy syndrome (HHE). Electroencephalogr Clin Neurophysiol Suppl (1982) 35:211–21. 6956495

[3] Auvin S Bellavoine V Merdariu D Delanoe C Elmaleh-Berges M Gressens P Hemiconvulsion-hemiplegia-epilepsy syndrome: current understandings. Eur J Paediatr Neurol (2012) 16(5):413–21. 10.1016/j.ejpn.2012.01.007 22341151

[4] Tenney JR Schapiro MB. Child neurology: hemiconvulsion-hemiplegia-epilepsy syndrome. Neurology (2012) 79(1):e1–4. 10.1212/WNL.0b013e31825dce5f 22753451

[5] Freeman JL Coleman LT Smith LJ Shield LK. Hemiconvulsion-hemiplegia-epilepsy syndrome: characteristic early magnetic resonance imaging findings. J Child Neurol (2002) 17(1):10–6. 10.1177/088307380201700103 11913562

[6] Auvin S Devisme L Maurage CA Soto-Ares G Cuisset JM Leclerc F Neuropathological and MRI findings in an acute presentation of hemiconvulsion-hemiplegia: a report with pathophysiological implications. Seizure (2007) 16(4):371–6. 10.1016/j.seizure.2007.01.009 17350294

[7] Mori Y Anatomopathology and pathogeny of the hemiconvulsion-hemiplegia-epilepsy syndrome. Part II. J Neurosurg Sci (1979) 23(1):1–22. 536746

[8] Berhouma M Chekili R Brini I Kchir N Jemel H Bousnina S Decompressive hemicraniectomy in a space-occupying presentation of hemiconvulsion-hemiplegia-epilepsy syndrome. Clin Neurol Neurosurg (2007) 109(10):914–7. 10.1016/j.clineuro.2007.07.027 17875361

[9] Toldo I Calderone M Boniver C Dravet C Guerrini R Laverda AM. Hemiconvulsion-hemiplegia-epilepsy syndrome: early magnetic resonance imaging findings and neuroradiological follow-up. Brain Dev (2007) 29(2):109–11. 10.1016/j.braindev.2006.06.005 16876973

[10] Sakakibara T Nakagawa E Saito Y Sakuma H Komaki H Sugai K Hemiconvulsion-hemiplegia syndrome in a patient with severe myoclonic epilepsy in infancy. Epilepsia (2009) 50(9):2158–62. 10.1111/j.1528-1167.2009.02175.x 19563349

[11] Yamazaki S Ikeno K Abe T Tohyama J Adachi Y. Hemiconvulsion-hemiplegia-epilepsy syndrome associated with CACNA1A S218L mutation. Pediatr Neurol (2011) 45(3):193–6. 10.1016/j.pediatrneurol.2011.04.010 21824570

[12] Scantlebury MH David M Carmant L. Association between factor V Leiden mutation and the hemiconvulsion, hemiplegia, and epilepsy syndrome: report of two cases. J Child Neurol (2002) 17(9):713–7. 10.1177/088307380201700914 12503653

[13] Mondal RK Chakravorty D Das S. Hemiconvulsion, hemiplegia, epilepsy syndrome and inherited protein S deficiency. Indian J Pediatr (2006) 73(2):157–9. 10.1007/BF02820211 16514228

[14] Bahi-Buisson N Kossorotoff M Barnerias C Boddaert N Bourgeois M Dulac O Atypical case of hemiconvulsions-hemiplegia-epilepsy syndrome revealing contralateral focal cortical dysplasia. Dev Med Child Neurol (2005) 47(12):830–4. 10.1111/j.1469-8749.2005.tb01089.x 16288674

[15] Laina I Syriopoulou VP Daikos GL Roma ES Papageorgiou F Kakourou T Febrile seizures and primary human herpesvirus 6 infection. Pediatr Neurol (2010) 42(1):28–31. 10.1016/j.pediatrneurol.2009.07.016 20004859

[16] Teach SJ Wallace HL Evans MJ Duffner PK Hay J Faden HS. Human herpesviruses types 6 and 7 and febrile seizures. Pediatr Neurol (1999) 21(4):699–703. 10.1016/S0887-8994(99)00068-5 10580881

[17] Kawada J Kimura H Yoshikawa T Ihira M Okumura A Morishima T Hemiconvulsion-hemiplegia syndrome and primary human herpesvirus 7 infection. Brain Dev (2004) 26(6):412–4. 10.1016/j.braindev.2003.12.003 15275707

[18] Ballif BC Rosenfeld JA Traylor R Theisen A Bader PI Ladda RL High-resolution array CGH defines critical regions and candidate genes for microcephaly, abnormalities of the corpus callosum, and seizure phenotypes in patients with microdeletions of 1q43q44. Hum Genet (2012) 131(1):145–56. 10.1007/s00439-011-1073-y 21800092

[19] Thierry G Beneteau C Pichon O Flori E Isidor B Popelard F Molecular characterization of 1q44 microdeletion in 11 patients reveals three candidate genes for intellectual disability and seizures. Am J Med Genet A (2012) 158A(7):1633–40. 10.1002/ajmg.a.35423 22678713

[20] Szklarczyk R Wanschers BF Nijtmans LG Rodenburg RJ Zschocke J Dikow N A mutation in the FAM36A gene, the human ortholog of COX20, impairs cytochrome c oxidase assembly and is associated with ataxia and muscle hypotonia. Hum Mol Genet (2013) 22(4):656–67. 10.1093/hmg/dds473 23125284

[21] Bourens M Boulet A Leary SC Barrientos A. Human COX20 cooperates with SCO1 and SCO2 to mature COX2 and promote the assembly of cytochrome c oxidase. Hum Mol Genet (2014) 23(11):2901–13. 10.1093/hmg/ddu003 24403053 PMC4014192

